# Fermentation Improves Calcium Bioavailability in *Moringa oleifera* leaves and Prevents Bone Loss in Calcium‐deficient Rats

**DOI:** 10.1002/fsn3.1653

**Published:** 2020-05-25

**Authors:** Jiahe Dai, Liang Tao, Chongyin Shi, Shuwen Yang, Depeng Li, Jun Sheng, Yang Tian

**Affiliations:** ^1^ College of Food Science and Technology Yunnan Agricultural University Kunming China; ^2^ Yunnan Provincial Key Laboratory of Biological Big Data Yunnan Agricultural University Kunming China

**Keywords:** bone mineral density, calcium absorption, microbial fermentation, *Moringa oleifera* leaf

## Abstract

Nowadays, there is an increasing demand of healthier plant calcium supplements. *Moringa oleifera* leaves (MOL) are rich in calcium and thus are promising candidates for developing efficient calcium supplements. Here, using fermentation‐based approaches, we developed a *Moringa oleifera* leaf ferment (MOLF), which contents higher levels of calcium. The therapeutic potential of the MOLF was also examined both in vitro and in vivo. Nine lactic acid bacteria and four yeasts were tested for better fermentation of MOL. Calcium‐deficient rats were used for evaluating the therapeutic effects of MOLF. The results of liquid fermentation showed that the mixture of *Lactobacillus reuteri, Lactobacillus acidophilus* *,* and *Candida utilis* elevated the content of MOL calcium most strikingly, with the content of calcium increased nearly 2.4‐fold (from 2.08% to 4.90%). The resulting MOLF was then subjected to cell experiments and animal experiments. The results showed that calcium absorption in Caco‐2 cells in MOLF group was higher than that in CaCl_2_ group significantly. Interestingly, in calcium‐deficient rats, MOLF treatment significantly increased the thickness of cortical bone, rat body weight, wet weight of the femur, and the femur bone density, whereas it decreased osteoclast numbers. These results indicate that microbial fermentation increased calcium bioavailability of MOL, promote the growth and development of calcium‐deficient rats, bone calcium deposition, and bone growth; enhance bone strength; reduce bone resorption; and prevent calcium deficiency.

## Graphical abstract

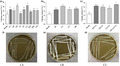

## 1 INTRODUCTION

Calcium is an important inorganic element in animals and human bodies, which is participated in the metabolism of the whole life, such as signal transmission, muscle contraction, and bone growth and was inseparable from the participation of calcium (Miller, Jarvis, & McBean, 2001). The amount of calcium in bones and teeth accounts for more than 99%, and the remaining 1% is present in intracellular and extracellular fluids (Nordin, 1997). Thus, insufficient calcium intake may be inevitably resulted in ailments of bones, including rickets and osteoporosis (Osborne et al., 1996). Noticeably, osteoporosis caused by calcium deficiency affects more than 75 million people worldwide. Even worse, the incidence of osteoporosis increased steadily in the world population, which is accompanied by significantly increasing social costs for treating osteoporosis (Kim & Park, 2013).

As early as more than 20 years ago, inorganic calcium supplements such as calcium carbonate and calcium chloride appeared on the market. However, the intestinal tract was an alkaline environment; inorganic calcium was liable to form insoluble calcium salts in this environment, resulting in a decrease in the body's ability to utilize calcium (Bronner & Pansu, 1999). Organic calcium, such as calcium gluconate and calcium lactate, occurred after the inorganic calcium. It is considered to be effective in promoting calcium absorption, because it did not require the participation of gastric acid during digestion, and it increased the solubility of minerals in the intestine (Sun et al., 2016). However, most of these chelates are costly and complex for preparation process (Goulart et al., 2014). What's more, these kinds of calcium products are mainly derived from animal products, indicating that these products may not fit the needs of vegetarians and people who are allergic to animal dairy (Peng, Hou, Zhang, & Li, 2017; Sun, Jin, Li, Yin, & Lin, 2017). From another point of view, plant‐based foods are more healthier (Rousseau, Kyomugasho, Celus, Hendrickx, & Grauwet, 2019). Therefore, there is an urgent need to increase the number of plant source calcium supplement products to provide more choices for consumers.

*Moringa oleifera* is native to India and a perennial deciduous tropical tree species. It is currently considered the most nutritious plant on the planet, as almost every part of the plant can be used as food or drugs (Lopez‐Teros et al., 2017). *Moringa oleifera* leaves (MOL) have been consumed by Asians as a health food for thousands of years. It has great nutritional and medicinal value, and can be used as a source of high‐quality protein. Noticeably, it is the plant with the highest calcium content, which is up to 2,500 ~ 3,000 mg/100 g, ~25 times higher than that in the milk, However, in the calcium absorption inhibitors in MOL, such as phytic acid, calcium cannot fully absorbed and utilized by the body. At present, the research of MOL mainly focused on the extraction of active ingredients. In addition, products related to fermented MOL have also appeared on the market. The main product types are that reduce fat, reduce sugar, and help sleep. Few studies have focused on the use of calcium in MOL.

Nutrient release is usually achieved by enzyme treatment, pH adjustment, or microbial fermentation. Microbial fermentation has become one of the basic methods of releasing nutrients. In the fermentation process, complex substances are broken down into smaller molecules by microorganisms, and nutritional quality and antiseptic properties are improved, and even new characteristics of healthy food can be obtained at the same time. Some microbial lactic acid bacteria related to fermented foods, like Bacillus, Aspergillus, and Yeast, can degrade phytic acid and other antinutritional substances by producing enzymes such as phytase and polyphenol oxidase, thereby improving the bioavailability of calcium (Hemalatha, Platel, & Srinivasan, 2007; Rousseau et al., 2019). Microbial fermentation was proved to be effective in improving the nutrient release of MOL (Miller et al., 2001). Also, recent studies have reported that the addition of *Moringa oleifera* leaf ferment (MOLF) to feed has a significant effect on improving tibia integrity and the inorganic components of the tibia bone in broiler chickens (Nkukwana, 2014). However, it is still unknown whether microbial fermentation could increase the release and bioactivity of calcium by MOL whether or not. On the other hand, it is still unknown how to fermentate the MOL to archive a higher nutritional value of MOL from the point of view of calcium.

Therefore, this study revealed the selection of strains suitable for the effective release of calcium and reduces antinutritional factors in MOLF by microbial fermentation, and combined cell experiments with animal experiments to explore the calcium bioavailability of MOLF and its impact on bone health. The completion of this work will help overcome the lack of nutrient release in the plant food matrix and the difficulty of calcium absorption, which may further promote the application of MOL in the plant calcium product market and increase the variety of plant calcium supplements.

## 2 MATERIALS AND METHODS

### 2.1 Raw materials and microorganisms

*Moringa oleifera* leaves was purchased from Yunnan Tianyou Technology Development Co., Ltd. Dried leaves of *Moringa oleifera* were chopped with an ultrafine grinder to obtain a powder and then fermented. The bacteria used for MOL fermentation are obtained from edible bacteria and are harmless to the human body. Thirteen strains (including 9 lactic acid bacteria and 4 yeasts) used in this experiment were obtained from the China Industrial Culture Research Center (Table 1).

**TABLE 1:** Thirteen strains and sources

Strain	Number
*Lactobacillus plantarum*	CICC 194165
*Lactobacillus casei*	CICC 6117
*Lactobacillus reuteri*	CICC 6226
*Lactobacillus johnsonii*	CICC 10861
*Lactobacillus bulgaricus*	CICC 6098
*Lactobacillus acidophilus*	CICC 6074
*Lactobacillus helveticus*	CICC 6032
*Lactobacillus rhamnosus*	CICC 6141
*Lactobacillus paracasei*	CICC 6270
*Saccharomyces cerevisiae*	CICC 1946
*Candida utilis*	CICC 1314
*Candida tropicalis*	CICC 1662
*Pichia kluyveri*	CICC 32845

### 2.2 Microbial fermentation

The weighed MOL was taken in a triangular flask and warm water (75 ~ 85°C) at a ratio 1:10 (w/v) added in. The flask was then placed on an electric furnace and heated to boiling. After boiling, the liquor was kept aside for 20 min, sealed with a sealing film, and then, the *Moringa oleifera* liquid was used after cooling.

On an aseptic table, 9 kinds of lactic acid bacteria and 4 kinds of yeasts (Table 1) domesticated with MOL (adapted for 10 generations) (Zhang et al., 2017) were added to the *Moringa oleifera* solution with an amount of 4% (v/v) of the added amount separately (the volume of the wet cells after acclimation accounted for MOL ferment broth proportional volume), evenly stirred, sealed, and then placed in an incubator at 36°C by shaking at 200 rpm for 72 hr for fermentation. The fermentation broth was centrifuged at 4,000 × *g* for 20 min, and then, the supernatant was collected. The optimal strain was determined by the experimental conditions in which the maximum amount of soluble calcium was produced by different strains. Lyophilization was performed for subsequent experiments to prepare the sample. The calcium contents were measured with an atomic absorption spectrophotometer (AA6300C, Shimadzu).

### 2.3 Establishment of calcium transport single‐layer model of Caco‐2 cells

A single‐layer model of calcium transport experiments was established using Caco‐2 cells (shanghai cell institute country cell bank, chain) with passage numbers between 30 and 60 passages (Hou, Liu, Shi, Ma, & He, 2017). The cells were cultured in DMEM medium containing 10% (v/v) fetal bovine serum, 1% (v/v) penicillin, and streptomycin, and the parameters of the cell culture incubator were set at 37°C and the CO_2_ concentration was 5%. The amount of cell inoculation was 1.2 × 10^5^ cells/ml into a 12‐well cell culture insert (well diameter, 0.4 µm; diameter, 12 mm; Coring Inc.), 1.5 ml laterally outside, apical side at 0.5 ml. The medium was replaced every 2 days for the first week, and the medium was changed every day after one week. To assess whether the Caco‐2 monolayer model was successfully established, transepithelial electrical resistance (TEER) was measured by using Millicell‐ERS system (Bronner & Pansu, 1999).

### 2.4 Calcium transport studies

When the TEER value is greater than 500 Ω × cm^2^, the cells monolayer model will be applied to this experiment (Hou et al, 2017). The cells were gently washed twice with a balanced salt solution of HBSS containing no calcium and magnesium, and 1.5 ml of buffer was added to the base chamber. Thereafter, MOL, MOLF broth, and 0.5 ml of 0.2 mg/ml calcium chloride having the same calcium concentration were added to the top and incubation was continued for 60 min. A 0.5 ml sample was taken from the basolateral side at 30 and 60 min for calcium content determination.

### 2.5 In vivo test of calcium absorption in rats

#### 2.5.1 Animals and diets

The animal experiment program was approved by the institutional animal Ethics Committee of Yunnan Agricultural University. Sixty male Sprague Dawley (*SD*) rats weighing 60–80 g (Peng et al., 2017) were obtained from Liaoning Changsheng Biotechnology Co., Ltd. [Certificate number: SCXK (Liao) 2015‐0001]. The rats were housed at a temperature of 22 ± 2°C, a relative humidity of 60 ± 5%, 12 hr of light, and 12 hr of darkness. All diets were purchased from Trophic Animal Feed High Tech Co., Ltd. and prepared according to the AIN‐93 diet. The calcium content in normal and low‐calcium feeds was 4,500 and 140 mg/kg, respectively.

After 7 days of adaptation, the rats were randomly divided into normal, model, and CaCO_3,_ and three experimental groups (the Ca^2+^ content of CaCO_3_ group and a moderate dose of MOLF with calcium content remaining equal) with rats in the low‐Ca, medium‐Ca, and high‐Ca groups were intragastric administration of 0.065, 0.13, and 0.195 g/kg of MOLF according to their body weight (calculated by calcium content) (*n* = 10/group). During the 4‐week feeding period, the control rats were given ad libitum access to the control diet, and the remaining experimental groups were given ad libitum access to low‐calcium feed. Both MOLF and calcium carbonate were dissolved in deionized water as a solvent (Peng et al., 2017).

During the experiment, the body weight of the rats was weighed weekly and the feed intake was recorded daily. The experiment was carried out for last 3 days of week 4 for conducting calcium metabolism experiments. In the last 3 days of the experiment, rat feces were collected and weighed daily, and calcium excreted in the feces was measured. The absorption rate calculation was done as follows:

$$\text{Intake calcium}\left(\text{mg}/d\right)=\text{calcium content in feed}\left(\text{mg}/g\right)\times \text{feed consumption}\;\left(g/d\right).$$

$$\text{Fecal calcium}\left(\text{mg}/d\right)=\text{fecal calcium content}\left(\text{mg}/g\right)\times \text{feces excretion}\left(g/d\right).$$

$$\text{Calcium apparent absorption rate}=\left(\text{intake of calcium}-\text{fecal calcium}\right)/\text{intake of calcium}\times 100\%.$$

### 2.6 Biochemical parameter analysis of blood samples

Four weeks after the rats were fed, all the rats were fastened for 12 hr, and blood was taken from the abdominal aorta. After being incubated for 1 hr at room temperature, the blood was centrifuged at 3,000 × *g* for 10 min at 4°C to obtain serum. Ca, Mg, P, and ALP in serum were measured based on the kit operating guidelines (Nanjing Institute of Bioengineering, China).

### 2.7 Determination of organ coefficient

The rats were sacrificed and dissected, and the heart, liver, spleen, lungs, and kidneys were taken out and weighed to calculate the organ index. The left and right femurs were anatomized, and the adherent tissue was eliminated. After the surrounding muscles were completely removed, the resulting femur weight was weighed. The organ coefficient was then calculated as follows: coefficient of each organ = weight of each organ/body weight, organ coefficient of wet femur = wet weight of the femur/body weight.

### 2.8 Detection of bone mineral density

After the rats were sacrificed, the left femur was removed, whose excess muscle and connective tissue were removed, and the femoral mineral density (BMD) was quickly determined using a dual‐energy X‐ray absorptiometry (DEXA).

### 2.9 Femur histological analyses

For histological analysis of rat right femur, after completely removing the surrounding muscles and tissues, the fresh femoral bone tissue was quickly collected, fixed in 10% formaldehyde for 48 hr, dehydrated with ethanol for 24 hr, and decalcified using EDTA. After decalcification was completed, paraffin was used for embedding, and the tissue was cut into 5 nm thick and stained with Trap, hematoxylin and eosin (H&E). The microscope was used for observation and images of bone tissue, estimated by medical image analysis system BI‐2000 (Taimeng).

### 2.10 SDS‐PAGE

Analysis of protein changes in MOLF was performed by SDS‐PAGE (12% separation gel, 4% concentration gel) to analyze the protein changes after MOLF. The electrophoresis conditions were as follows: 50 V interval gel for 30 min and a 120 V separation gel for 80 min. Next, the sample was stained with Coomassie Brilliant Blue (G‐250, Bio‐Rad). The dyed tape was then exposed using an automated imaging system (Protein Simple).

### 2.11 Analytical methods

The amino acid composition of MOLF was analyzed using an Agilent 1,200 HPLC system (Agilent Technologies) and a sulfonic acid type cationic resin separation column (4.6 mm × 60 mm × 33 µm) (Gonzalez‐Vega, Kim, Htoo, Lemme, & Stein, 2011).

Two grams of fermentation product was used to determine the protein concentration of the 72‐hr sample. The sample was dissolved at a ratio of 1:10 (W/V), centrifuged, and precipitated, and the protein content in the supernatant was measured using a BCA Quantitation Kit (Sigma). Absorbance values were measured at 560 and 680 nm (UNICO UV‐2000).

### 2.12 Determination of peptide yield

The peptide yield was determined according to trichloroacetic acid (TCA) method. Next, 2.5 ml of 10% TCA was added to 2.5 ml of the growth product. It was allowed to stand at room temperature for 20 min and centrifuged at 4,000 × *g* for 15 min. The content of the peptide in the sample was determined by a biuret method, using a tetrapeptide Gly‐Gly‐Tyr‐Arg as a standard liquid. The content of oxalic acid and lactic acid was determined by ion chromatography (Levart, 2000).

### 2.13 Statistical analysis

The experimental data were statistically analyzed using GraphPad Prism 5 software (GraphPad Software). All indicators were expressed as mean ± *SEM*. Comparisons between groups analyzed using one‐way ANOVA and followed by Tukey–Kramer post hoc test and independent‐sample *t* test. *p* < .05 was considered to be statistically significant.

## 3 RESULTS AND DISCUSSION

### 3.1 Strain screening

**FIGURE 1:**
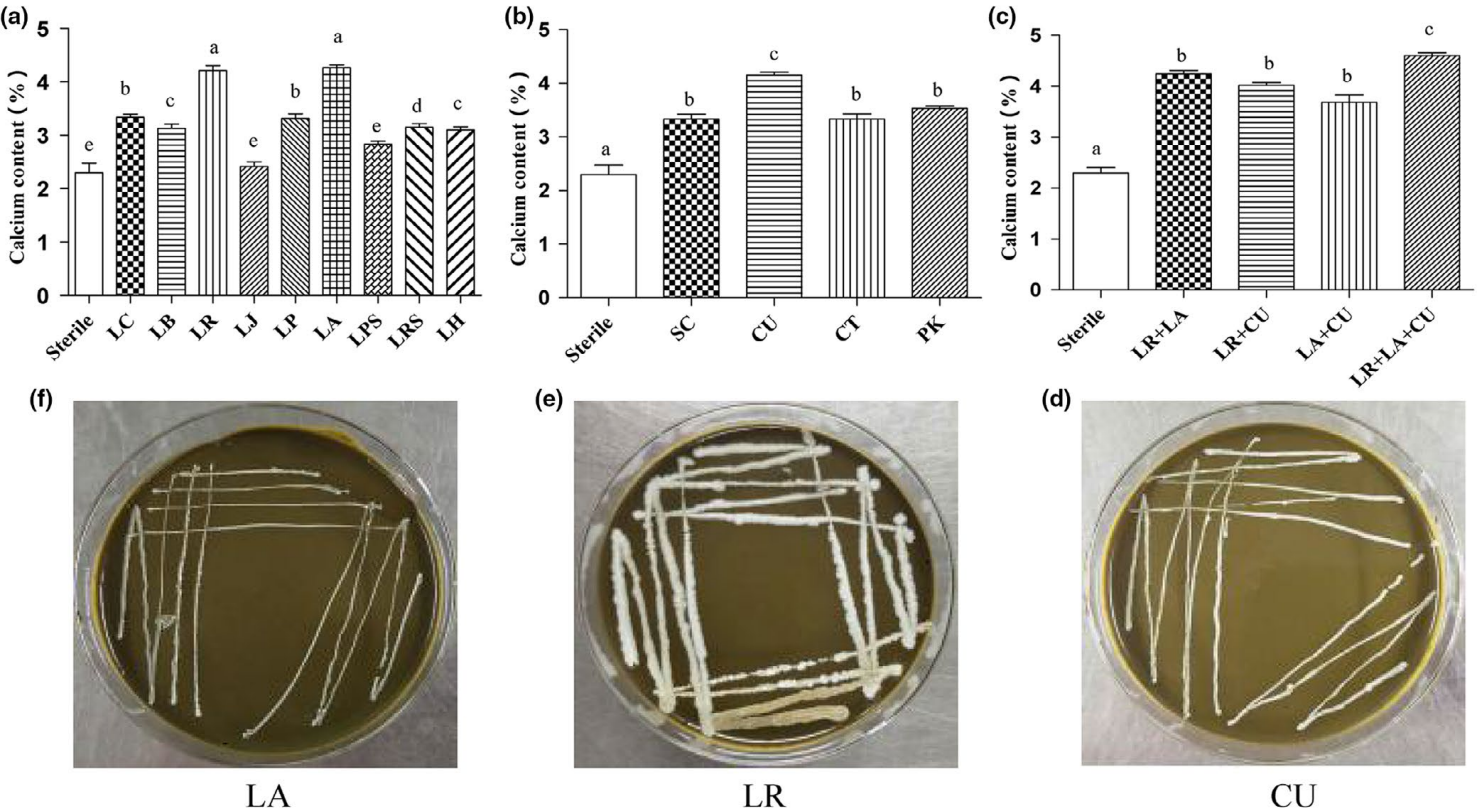
The effect of different microorganisms on calcium content. (a) Represents 9 kinds of lactic acid bacteria, (b) represents 4 kinds of yeast, and (c) represents mixed‐strain ferment calcium content in samples containing 10% (w/v) MOL for 72 hr of solid matter, and (d) is the growth of *Lactobacillus acidophilus* (LA), (E) is *Lactobacillus reuteri* (LR), and (f) is *Candida utilis* (CU). Medium containing 10% (w/v) *Moringa oleifera* acts as the sole carbon source at 37°C. All assays were performed in triplicate. *Lactobacillus plantarum*, *Lactobacillus casei*, *Lactobacillus reuteri*, *Lactobacillus johnsonii*, *Lactobacillus bulgaricus*, *Lactobacillus acidophilus*, *Lactobacillus helveticus*, *Lactobacillus rhamnosus*, *Lactobacillus paracasei*, *Saccharomyces cerevisiae*, *Candida utilis*, *Candida tropicalis*, and *Pichia kluyveri* are represented by LP, LC, LR, LJ, LB, LA, LH, LRS, LPS, SC, CU, CT, and PA, respectively

Nowadays, fermented foods have eating habits in most countries of the world due to their ease of storage, unique taste and aroma, and health benefits (Holzapfel, 2002). To screen the strains that increased the calcium content in MOL, the effects of fermentation on calcium content in MOL were evaluated. The calcium content after fermentation was used as indicator. Compared to fermentation with other lactic acid bacteria, the results after fermentation with *Lactobacillus reuteri* and *Lactobacillus acidophilus* demonstrated a higher calcium content of the fermented material (*p* < .05) (Figure 1a). However, there is no significant difference between the two types of lactic acid bacteria. Therefore, the subsequent experiments need to consider the effect of both types of lactic acid bacteria on the calcium content in the fermented extract. To increase the absorption efficiency, four common edible yeasts were also used to ferment MOL and observed the effects of yeast on calcium content. The results are shown in Figure 1b, in which the calcium content was higher in *Candida utilis* than other groups, 4.15%. Therefore, *Candida utilis* was identified as a potential available yeast.

In the long‐term research and production practice, the fermentation of single strains has shown the limitations of the scope of application, such as the fermentation process with complex ingredients or complex biochemical processes that cannot be completed well. However, mixed fermentation can complement each other because of its advantages, which opens a new chapter for solving the limitation of single‐strain fermentation (Sudun, Arakawa, Miyamoto, & Miyamoto, 2012). At present, in the food field, there are more and more products fermented by lactic acid bacteria and yeast. The products fermented by mixed bacteria have shown higher quality than the products fermented by a single strain in terms of taste, flavor, nutritional value, and physiological function. In addition to giving the product a unique flavor, yeasts can also form a symbiotic effect with lactic acid bacteria (Freire, Ramos, & Schwan, 2015). Yeast provides many nutritional factors for lactic acid bacteria, such as amino acids and vitamins. The metabolites of lactic acid bacteria provide energy sources for yeast (Roostita, 1996). Therefore, the combined fermentation of lactic acid bacteria and yeasts was conducted to study the effect on calcium content. The *Lactobacillus reuteri*, *Streptococcus thermophilus*, and *Candida utilis* strains were combined with *Lactobacillus reuteri* + *Lactobacillus acidophilus*, *Lactobacillus reuteri* + *Candida utilis*, *Lactobacillus acidophilus* + *Candida utilis*, and *Lactobacillus reuteri* + *Lactobacillus acidophilus* + *Candida utilis*, and used for fermentation. As shown in (Figure 1c), the combination of *Lactobacillus acidophilus* + *Candida utilis* + *Lactobacillus reuteri* had the highest calcium content. The calcium content was increased from 2.08% to 4.90%, which effectively increased the calcium content (*p* < .05). Three strains grew well in the MOL medium (Figure 1d–f).

It has been studied that many plant foods are rich in minerals including calcium and zinc, but it is difficult for them to completely release during digestion, thus limiting absorption (Platel & Srinivasan, 2016). As shown in Figure 1, calcium was effectively released from MOL after mixed fermentation by microorganisms, the possibility of promoting calcium absorption. Although the content of calcium in MOL is high, but it is not in a free state, and it often exists in complex and insoluble salt forms with phytic acid and oxalic acid, or chimeric in macromolecular polysaccharides. When leaching with water, the calcium in MOL is difficult to escape. After fermentation by lactic acid bacteria, it can metabolize large molecular sugars, release calcium, increase the extraction rate, and, at the same time, produce lactic acid and further form calcium lactate with calcium (Figure 1a), increasing calcium absorption. Yeast fermentation can also increase the calcium content in the extract (Figure 1b). The main reason is that the yeast is a facultative anaerobic bacteria, which has a strong ability to produce enzymes. Protease and phytase can be produced during the fermentation process, which will further degradation of proteins and phytic acid, releasing calcium ions (Vicente‐Soler, Arguelles, & Gacto, 1991). This is consistent with Coda's research that microbial fermentation can reduce the phytic acid content in wheat bran (Coda, Rizzello, Curiel, Poutanen, & Katina, 2014). Lactic acid bacteria can be symbiotic with yeast. Yeasts multiply rapidly in the prefermentation and dominate. However, with the proliferation of yeast, metabolites such as pyruvate and succinic acid can stimulate the metabolic activities of lactic acid bacteria. Lactic acid bacteria began to proliferate and metabolize in large quantities (Leroi & Pidoux, 1993), which produced a large amount of lactic acid. Phytic acid was more easily degraded in this environment (Buddrick, Jones, Cornell, & Small, 2014). At the same time, the coordinated fermentation of yeast and lactic acid bacteria plays an important role in improving the flavor and nutritional composition of the product, which is more conducive to the development of potential calcium supplement products (Wang, Hou, & Cao, 2011).

### 3.2 Changes in the main influential factors during calcium absorption before and after fermentation

Studies have reported that a low protein diet can decrease calcium absorption (Kerstetter, O’Brien, & Insogna, 2001). Calcium and protein can be combined, which prevents calcium from precipitating during digestion, thereby improving calcium absorption. Adequate protein intake is regarded as good for bone health (Erba, Ciappellano, & Testolin, 2001; Vavrusova, Raitio, Orlien, & Skibsted, 2013). In addition, due to the strong affinity between calcium and protein, peptides, and certain amino acids, calcium can also form a chelate with some amino acids and polypeptides. These can prevent the decomposition and loss of calcium to a certain extent, thereby preventing the formation of precipitates during digestion (Tang & Skibsted, 2016).

**FIGURE 2:**
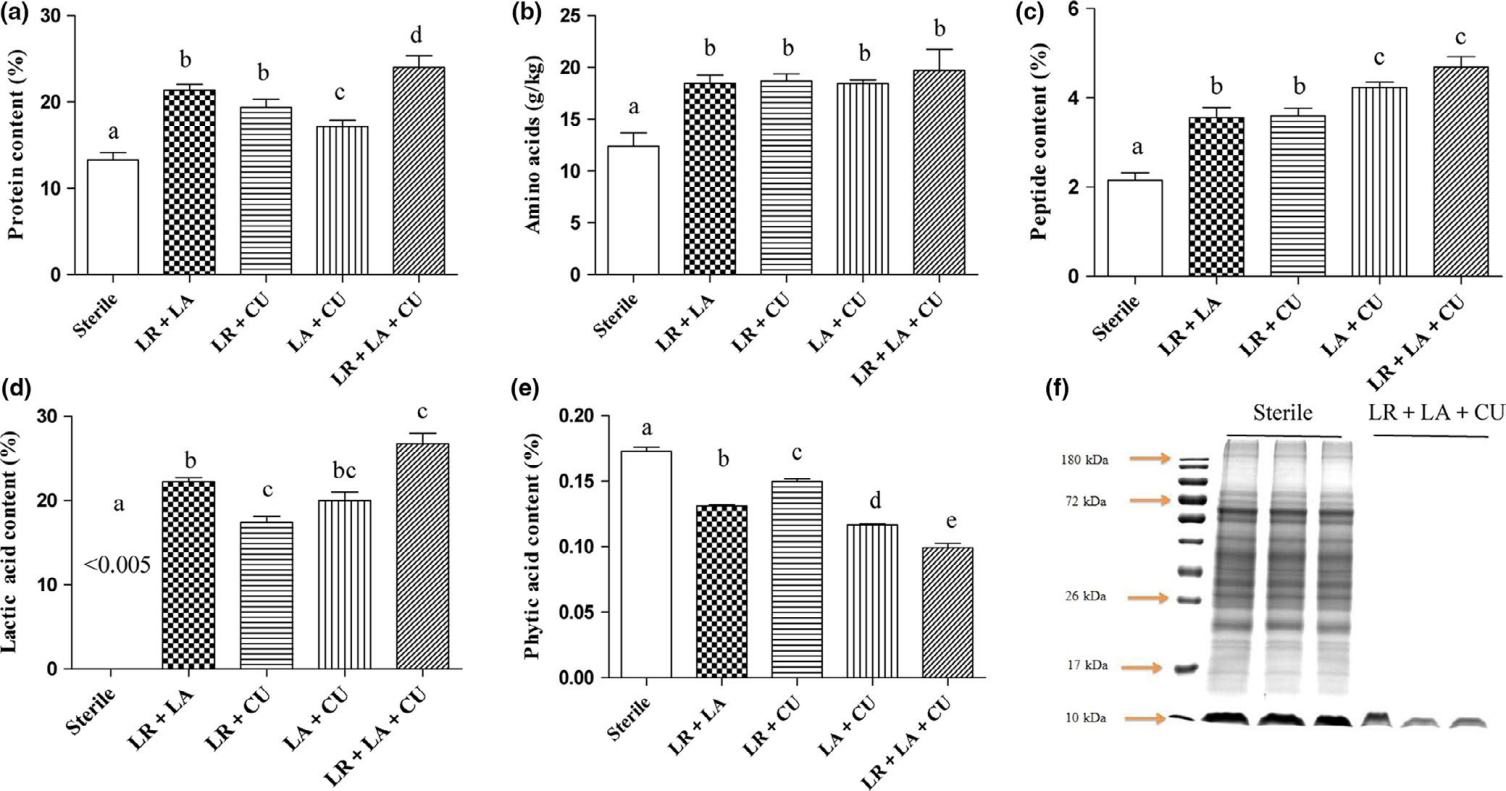
The changes of calcium content (a), protein content (b), total amino acid content (c), polypeptide content (d), and phytate content (e) before and after fermentation of MOL using different strain combinations. (f) SDS‐PAGE before and after fermentation of MOL using LR+LA+CU. Track 0, marked. Lanes 1–3 represent electrophoretic bands of protein in unfermented MOL. Lanes 4–6 represent electrophoresis bands of proteins in MOLF

In order to further evaluate and screen the optimal strain combination, the nutritional composition of MOL was measured after fermentation with *Lactobacillus reuteri* + *Lactobacillus acidophilus*, *Lactobacillus reuteri + Candida utilis*, *Lactobacillus acidophilus* + *Candida utilis*, and *Lactobacillus reuteri* + *Lactobacillus acidophilus* + *Candida utilis*. Nutrition changes are shown in Figure 2a, and the four combined fermentations can increase the water‐soluble protein content, but the three bacteria combined fermentation have the highest content, which is significantly higher than the other groups (*p* < .05), increasing from 13.3% to 24.1%. The measurement results of free amino acid content showed that there were no significant differences between the fermentation groups, but they were significantly higher than those of the nonfermented group (*p* < .05), and the content of amino acid increased from 12.41 to 19.7 g/kg (Figure 2b). This shows that microbial fermentation can increase the content of free amino acids. The changes of peptides are similar to the changes of soluble proteins, and compared with the two bacteria combined fermentation group, the peptide content produced by the three bacteria combined fermentation was the highest, which increased from 2.04% to 4.90% (Figure 2c). These results were similar to those found in Zhang et al. study (2017). Microbial fermentation can release nutrients in MOL. Increase in the content of small molecular proteins and amino acids might be beneficial for increasing calcium bioavailability.

Phytic acid is a well‐known mineral antinutrient, which is one of the antinutrients in MOL that associates with calcium to form phytate complex precipitates. Due to lack of endogenous phytase, these phytate complexes are difficult to digest by gastric animals such as humans (Hurrell & Egli, 2010). Therefore, the bioavailability of calcium is lowered. At the same time, the presence of phytic acid also has an adverse effect on the absorption and utilization of proteins, vitamins, and minerals. Therefore, the change in phytic acid content demonstrated a great significance in evaluating the effect of calcium absorption. Also, it is exciting that the content of phytic acid after fermentation was significantly reduced, and the three bacteria‐mixed fermentation group was significantly lower than the other groups (*p* < .05), from 0.172% to 0.098%, which was 1.7 times lower (Figure 2e). The results of this were also similar to the results reported by Rase et al, in which three major microorganisms were used during the fermentation of legumes and grains: lactic acid bacteria, Bacillus, Aspergillus, and yeast. Some microorganisms that showed association with fermented foods are capable of producing enzyme phytase, which in turn degrades antinutritional components such as vegetable acids (Raes, Knockaert, Struijs, & Van Camp, 2014). This result also suggested that the reduction in phytic acid resistance to nutrient absorption factor after fermentation might contribute to the increase in calcium bioavailability during the fermentation.

The reduction of phytic acid in addition to the effect of phytase also has the effect of acid. Under acidic conditions, phytic acid is more easily degraded (Buddrick et al., 2014). Yeast fermentation can produce acid, and lactic acid bacteria are more acid‐producing than yeast. Lactic acid bacteria fermentation produces a large amount of lactic acid, which reduces the pH value of the fermentation broth, which is beneficial to maintaining endogenous phytase activity. Phytic acid is rapidly hydrolyzed by enzymes under acidic conditions (Konietzny & Greiner, 2002). The acidic environment leads to the extraction of hydride ions and the rearrangement of phenolic structures, affecting the complexity of antinutritional components in minerals (Towo, Matuschek, & Svanberg, 2006). Organic acids can form soluble and absorbable ligands with minerals, preventing the formation of insoluble complexes and increasing the bioavailability of minerals (Sokrab, Mohamed, & Babike 2014). For example, lactic acid and calcium form calcium lactate, which is conducive to the absorption and transport of calcium. As shown in Figure 2d, the lactic acid content before fermentation was less than 0.005%, and the lactic acid content after fermentation was as high as 26.8%. These results show that fermentation can release the nutrients in MOL and reduce the antinutritional factors, which will help promote the absorption and transport of calcium, and the fermentation effect of the three bacteria combination is the best. Further examining molecular weight distribution of proteins in MOL unfermented and mixed fermentation with three bacteria, it was found that although the protein content increased after fermentation, the distribution of protein molecular bands after fermentation was less, mainly concentrated at 10 kDa and below (Figure 2f). It may be that the protein after the increase in fermentation is rapidly decomposed into small molecule peptides and amino acids by the protease of microbial metabolism. The increase in small molecule protein and amino acid content may help increase calcium bioavailability. The results indicated that calcium absorption will be beneficial after fermentation. As mentioned above, MOLF for subsequent in vitro and in vivo testing will be prepared by using three mixed bacteria.

### 3.3 The calcium transport study in vitro

**FIGURE 3:**
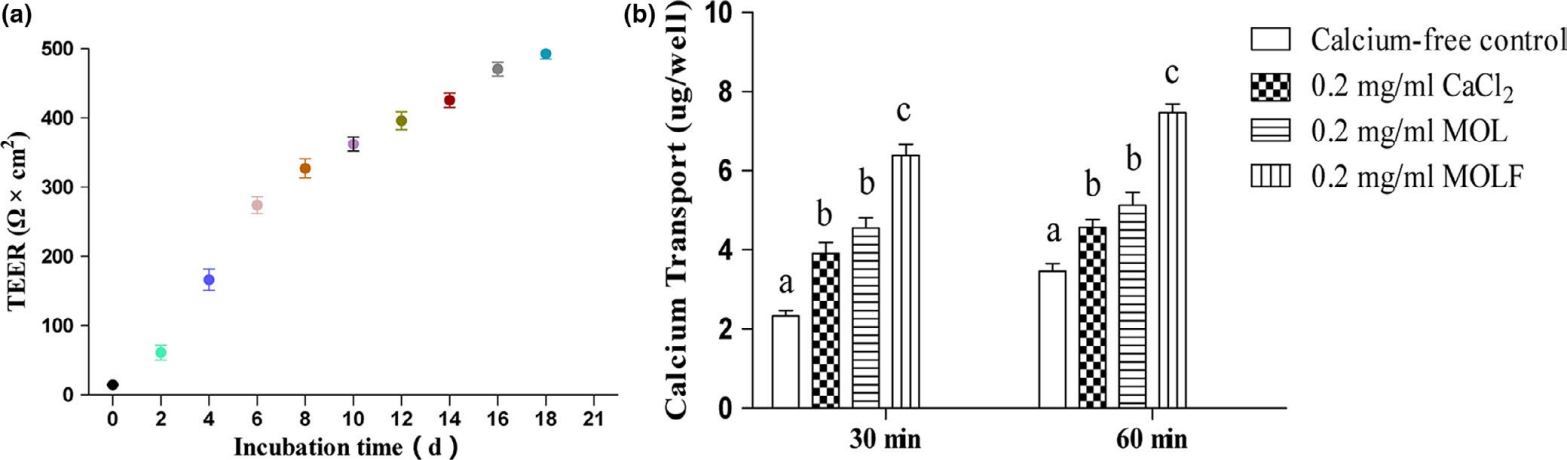
Calcium absorption studies. a, Establishment of Caco‐2 cell monolayer model. Cells were seeded on 12‐well transwell culture plates. Transepithelial electrical resistance (TEER) was determined every other day by using a Millicell‐ERS system to assess the integrity of Caco‐2 cell monolayers. b, Calcium transport studies. The monolayers were then moved to a new plate containing 1.5 ml of HBSS buffer. 2 mg/ml CaCl_2_ or 2 mg/ml MOL or MOLF in 0.5 ml HBSS buffer (pH 7.4) was added to the apical side and incubated at 37°C for 1 hr. Next, 1.0 ml of sample was extracted from the basolateral side at 30 and 60 min, and calcium contents were determined by atomic absorption spectrophotometer. Different letters indicate significant difference among the groups at the given time point (*p* < .05)

The Caco‐2 monolayer cell model has been widely used for in vitro calcium absorption and other mineral absorption studies. By measuring the TEER value of the cells, we found that the TEER value increased over the incubation time by more than 500 Ω × cm^2^ on the 21st day, as shown in Figure 3a. This was similar to the study of Hou et al. (2015). This finding indicates the integrity of the monolayer, which means that the model can be used for calcium absorption experiments.

After cell monolayer was formed, MOL and MOLF with the same calcium concentration of 2 mg/ml were added to the upper room, the calcium content on the basolateral compartment was measured, and 2 mg/ml CaCl_2_ was used as a control. The results are shown in Figure 3b. Calcium and CaCl_2_ in MOL revealed that MOLF demonstrated higher calcium transport capacity in monolayer, compared to calcium‐free control (*p* < .05). At 120 min of incubation, MOLF showed a significant increase in calcium transportation when compared to 2 mg/ml CaCl_2_ and 2 mg/ml MOL (*p* < .05). In particular, the 2 mg/ml MOLF showed the highest calcium transport capacity, compared to calcium‐free control value (*p* < .05). These results indicated that the absorption of calcium in vitro can be improved after fermentation. This result was similar to the study of Lestienne et al., wherein the bioavailability of mineral elements can be effectively increased through the reduction and removal of phytic acid (Lestienne, Besancon, Caporiccio, Lullien‐Pellerin, & Treche, 2005). Therefore, in the next animal experiments, mainly the calcium bioavailability of MOLF and its effect on bone mineral density and femur characteristics in calcium‐deficient rats were studied.

### 3.4 Effect of MOLF on calcium absorption in *SD* rats

During feeding, the rats demonstrated no abnormal conditions, such as death and diarrhea. Table 2 shows the calcium intake during the last 3 days, apparent absorption rate and retention rate of calcium in different groups. Due to lack of calcium intake in the low‐calcium group, it was in a state of “calcium starvation,” and the absorption rate of calcium was significantly higher than that of other experimental groups, and this finding was similar to that of Welch and Hardcastle (2014). With the increased of calcium intake in MOLF, the absorption rate and storage rate of calcium were decreased gradually. The absorption rate and storage rate of calcium in the normal feeding group and the high‐dose group of MOLF group were significantly lower than that of the other groups. However, compared with the CaCO_3_ group, the absorption rate and retention rate of calcium in the medium‐dose MOLF group (same as the Ca content in the CaCO_3_ group) were significantly improved (*p* < .05). The absorption of calcium in the digestive tract mainly includes two pathways of active transport and passive diffusion. Active transport relies on the “pump protein” on the cell membrane, which is a kind of reverse concentration gradient transport, which requires energy consumption. When the calcium intake is low, active transport accounts for the main part of calcium absorption. Passive diffusion relies on the difference in osmotic pressure and solute concentration on both sides of the membrane, and it follows the concentration gradient without energy consumption. When the calcium intake is high, passive diffusion accounts for the main part of calcium absorption. Active transport mainly occurs in the duodenum, and the calcium absorption rate is high, while passive diffusion mainly occurs in the large intestine, and the absorption rate of calcium is less than 10% (Bronner & Pansu, 1999). Therefore, when the calcium intake is low, active calcium transport accounts for the vast majority, and the absorption rate of calcium rises rapidly, which increases the absorption rate of calcium. However, when the calcium intake is high, the active absorption of calcium tends to be saturated, the passive diffusion takes up the main part, and a calcium absorption platform effect will occur, leading to a decrease in the calcium absorption, and the calcium absorption and intake are in log negative correlation (Heaney, Weaver, & Fitzsimmons, 1990). This result indicated that the MOLF can promote calcium absorption in rats when the calcium content is 0.065 and 0.13 mg/kg, and the effect was better than the effect in the CaCO_3_ control group.

**TABLE 2:** Calcium absorption rates of normal and calcium‐deficient rat after different Ca treatments

Calcium absorption	Normal	Low‐Ca	M‐Low‐Ca	M‐Medium‐Ca	M‐High‐Ca	CaCO_3_
Calcium intake (mg/day)	62.1 ± 4.56	5.65 ± 0.16	27.12 ± 0.28	48.86 ± 0.52	70.90 ± 1.30	49.02 ± 0.31
Ca absorption (%)	62.16 ± 1.58^d^	91.03 ± 1.21^a^	83.19 ± 0.74^b^	80.72 ± 1.24^b^	65.71 ± 1.60^cd^	72.83 ± 3.29^c^
Ca retention (%)	58.67 ± 1.86^e^	84.05 ± 2.89^a^	78.35 ± 0.94^b^	77.95 ± 1.11^b^	62.89 ± 1.38^d^	71.15 ± 1.39^c^

Note

Data are expressed as mean ± standard deviation (*n* = 10). Any two means in the same row followed by the same letter are not significantly different (*p* > .05)

### 3.5 Serum levels of Ca, P, Mg, and ALP

**FIGURE 4:**
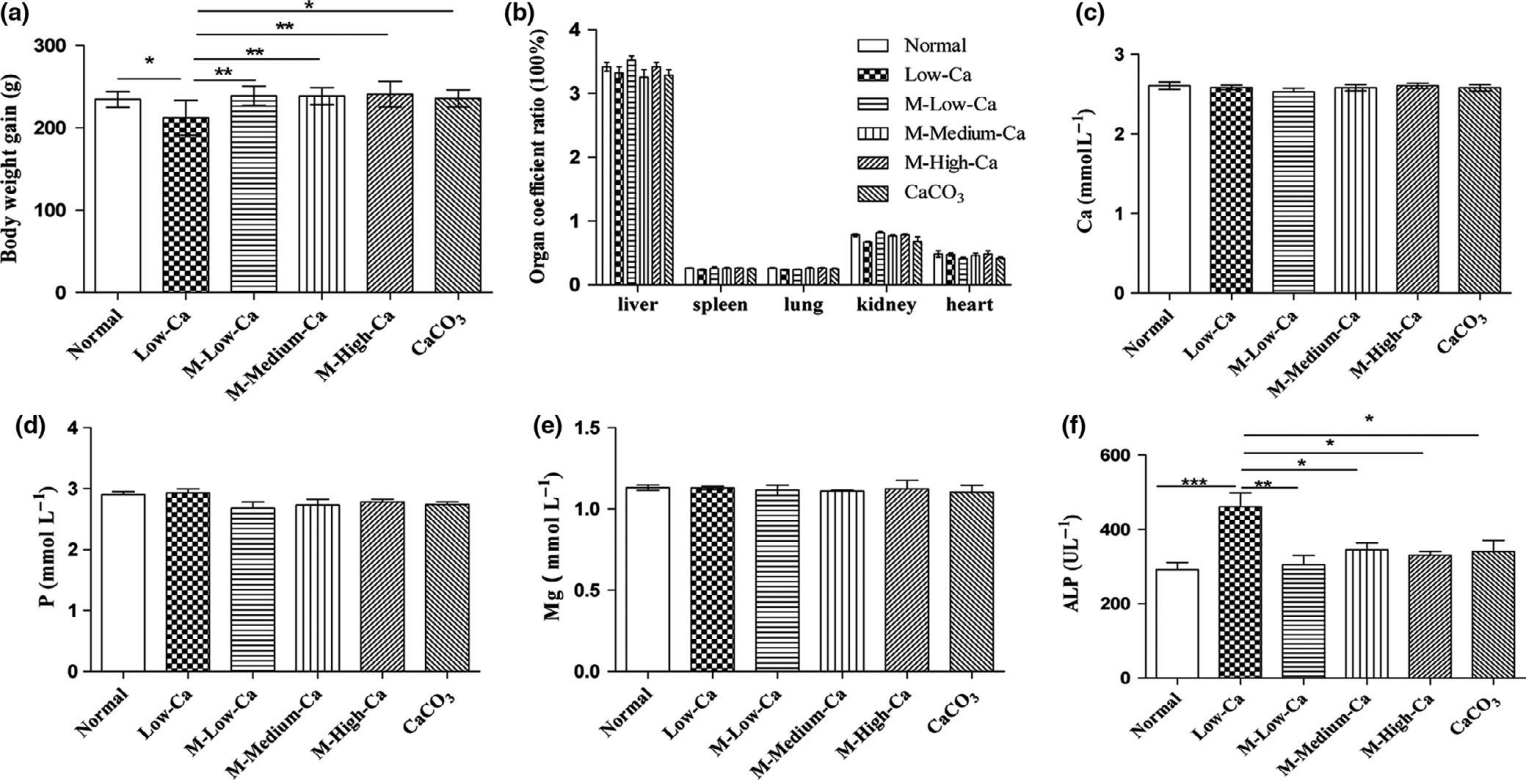
Weight gain in different groups (a) during feeding, and visceral index in different groups (b) in different organs. The levels of serum calcium (c), phosphorus (d), magnesium (e), and ALP (f). The rats in the control group were fed with normal diet ad libitum. The model group rats were fed with low‐calcium diet at random, M‐Low‐Ca, M‐Medium‐Ca, and M‐High‐Ca groups daily with 0.065, 0.13, and 0.195 g/kg body weight/day spicy. The extract (calculated by calcium content) at 4 weeks was calculated, followed by administration of low‐calcium diet. All data are expressed as mean ± *SEM* (*n* = 10). *p < .05 and **p < .01 compared with calcium‐deficient group. #*p* < .05 and ##*p* < .01 compared with CaCO_3_ group

After 4‐week feeding period, the weight gain of the rats was much lower in the low‐Ca group than in the remaining five groups (Figure 4a), and the visceral index data of the different groups showed no significant differences (Figure 4b). No obvious abnormalities were observed in any of the organs during the anatomical process. This preliminarily indicated that after fermentation, MOLF could promote the growth of calcium‐deficient rats, with no obvious side effects on rats, and hence was considered safe.

There was no significant difference in serum Ca, P, and Mg levels between the experimental groups (Figure 4c–e). The Ca content in the MOLF was increased, and the serum Ca level showed no change. This result was similar to that reported by Liu et al. (2017), and the decrease in serum Ca level in rats after feeding on MOLF might be due to massive deposition of blood Ca in the bones. This in turn demonstrated an increasing trend in the femur weight of the experimental group, and the femur bone density was significantly higher in the experimental group than in the low‐calcium group. ALP showed a close association with the bone calcification. ALP action in osteoblasts produced phosphoric acid, and Ca‐phosphate was deposited in bone. There was a significantly increase in ALP activity when the Ca intake was insufficient. ALP activity was significantly reduced (Figure 4f, *p* < .05). This result was similar to that reported by Chen et al. (2014). They found that the effects of desalted duck egg white peptide and tilapia skin calcium–peptide complex on Ca absorption in rats were observed in serum during long‐term low‐Ca intake. Also, the level of alkaline phosphatase activity was enhanced.

### 3.6 Femur characteristics

**FIGURE 5:**
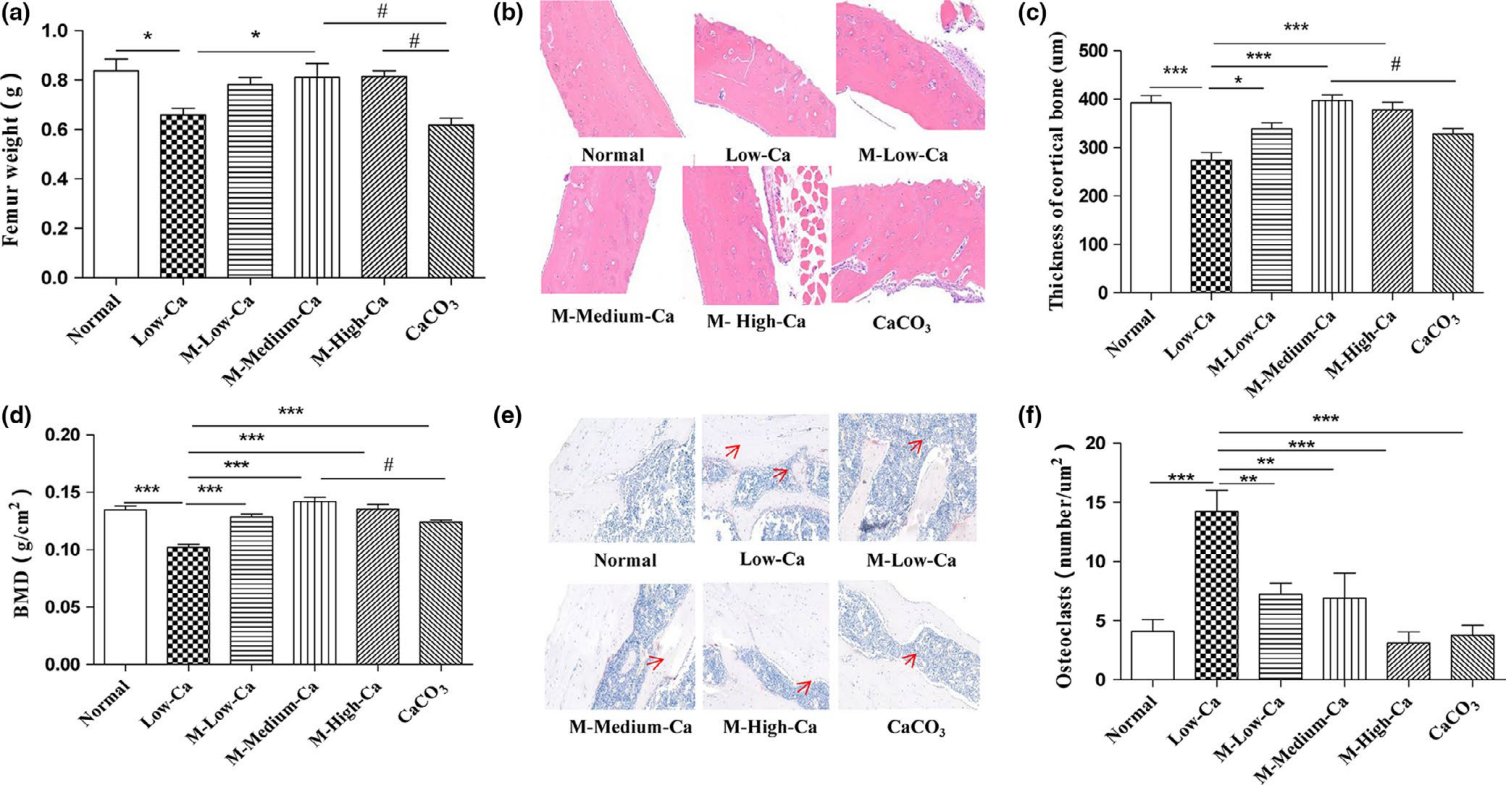
Femur features. Wet weight of the femur in each group (a), bone mineral density (BMD) (b). Cortical bone tissue was stained with hematoxylin and eosin (H&E) (c), and cortical bone thickness (d) was calculated. Osteoclasts were stained with Trap (e), and the number of osteoclasts (f) was calculated. A representative image was acquired using a medical image analysis system, and the original magnification was ×200. All data are expressed as mean ± *SEM* (*n* = 10). *p < .05 and **p < .01 compared with calcium‐deficient group. #*p* < .05 and ##*p* < .01 compared with CaCO_3_ group

As shown in Figure 5a, at the end of the feeding period, the wet weight of the femur in the MOLF dose group was higher than the calcium deficiency group (*p* < .05) and was higher than the same dose of CaCO_3_ group (*p* < .05), significantly. The femoral weight in other groups was higher than calcium deficiency group (*p* < .05). In addition, the BMD of the femur in each group was significantly higher than calcium‐deficient group, and the middle‐dose MOLF group (same calcium content as the CaCO_3_ group) was higher than CaCO_3_ group (*p* < .05), which was similar to the normal group (Figure 5b). Through bone tissue sectioning, by feeding the MOLF, the cortical bone thickness of the low‐calcium rat showed a significant increase (Figure 5c, d), and the number of osteoclasts was decreased (Figure 5e, f). This result was similar to some of the previously reported calcium supplements and fermented foods that promoted bone health outcomes (Ikeda et al., 2006; Nirmala et al., 2019). MOLF explained can significantly promote the growth of rats and promote calcium deposition and bone growth in rat bones, improving the bone strength. This might be mainly due to the effective release of calcium and vinegar calcium in the moringa, and the reduction of antinutrition absorption inhibitors, which thereby enhances the absorption of calcium and retention of calcium, and prevents calcium loss.

## 4 CONCLUSIONS

In this study, by screening the strains, the mineral elements like calcium and nutrients in MOL were released, and the antinutrient absorption factor phytic acid was reduced. Calcium bioavailability in MOLF was systematically assessed using an in vitro Caco‐2 cell monolayer model and an in vivo *SD* rat animal experiment. At 30 and 60 min, calcium in MOLF was effectively absorbed, compared to other groups. In the animal experiment, compared with control group, MOLF significantly increased the femur weight, bone density, cortical bone thickness, and decreased the number of osteoclasts in the femur and decreased serum ALP content. These findings suggested the potential effects of MOLF in improving the bone formation and preventing bone resorption. MOL are rich in calcium, but few studies have been made on the use of calcium in it. This discovery provides new ideas for the study of the mineral elements of MOL. The results also indicate that MOL may be a potential plant material for calcium supplements. This research will also inject a new energy into the calcium product market. In future, the exact mechanism of MOLF for calcium absorption and bone formation should be further explored.

*Moringa oleifera* leaf ferment effectively releases calcium and increases calcium bioavailability to prevent bone loss in rats with calcium deficiency during development. Therefore, MOLF is considered as a potential new product of plant calcium supplements.

## CONFLICT OF INTEREST

The authors declare that they have no conflict of interest.

## ETHICAL STATEMENTS

The animal experiment program was approved by the institutional animal Ethics Committee of Yunnan Agricultural University.
